# Etiology of Coinfections in Children with Influenza during 2015/16 Winter Season in Nepal

**DOI:** 10.1155/2018/8945142

**Published:** 2018-10-28

**Authors:** Bishnu Prasad Upadhyay, Megha Raj Banjara, Ram Krishna Shrestha, Masato Tashiro, Prakash Ghimire

**Affiliations:** ^1^Central Department of Microbiology, Tribhuvan University, Kirtipur, Nepal; ^2^National Public Health Laboratory, Department of Health Services, Kathmandu, Nepal; ^3^National Institute of Infectious Disease, Tokyo, Japan

## Abstract

Acute respiratory infections (ARIs) are one of the major public health problems in developing countries like Nepal. Besides the influenza, several other pathogens are responsible for acute respiratory infection in children. Etiology of infections is poorly characterized at the course of clinical management, and hence empirical antimicrobial agents are used. The objective of this study was to characterize the influenza and other respiratory pathogens by real-time PCR assay. A total of 175 throat swab specimens of influenza-positive cases collected at National Influenza Center, Nepal, during the 2015/16 winter season were selected for detecting other respiratory copathogens. Total nucleic acid was extracted using Pure Link viral RNA/DNA mini kit (Invitrogen), and multiplex RT-PCR assays were performed. Influenza A and B viruses were found in 120 (68.6%) and 55 (31.4%) specimens, respectively, among which coinfections were found in 106 (60.6%) specimens. Among the influenza A-positive cases, 25 (20.8%) were A/H1N1 pdm09 and 95 (79.2%) were A/H3 subtypes. Viruses coinfected frequently with influenza virus in children were rhinovirus (26; 14.8%), respiratory syncytial virus A/B (19; 10.8%), adenovirus (14; 8.0%), coronavirus (CoV)-HKU1 (14; 8.0%), CoV-OC43 (5; 2.9%), CoV-229E (2; 1.1%), metapneumovirus A/B (5; 2.9%), bocavirus (6; 3.4%), enterovirus (5; 2.9%), parainfluenza virus-1 (3; 1.7%), and parainfluenza virus-3 (2; 1.1%). Coinfection of *Mycoplasma pneumoniae* with influenza virus was found in children (5; 2.8%). Most of the viral infection occurred in young children below 5 years of age. In addition to influenza virus, nine different respiratory pathogens were detected, of which coinfections of rhinovirus and respiratory syncytial virus A/B were predominantly found in children. This study gives us better information on the respiratory pathogen profile and coinfection combinations which are important for diagnosis and treatment of ARIs.

## 1. Introduction

ARIs are one of the major causes of mortality and morbidity in children especially in developing countries [1]. The World Health Organization (WHO) estimated that 1.9 million children die every year due to respiratory tract infections (RTIs), mainly pneumonia in African and Southeast Asian countries [2]. The incidence of influenza-like illness (ILI) is almost similar between developed and developing countries like Nepal; the mortality rates are higher in developing countries. The frequency of mixed infections of noninfluenza ARI viruses with influenza virus varies from 10 to 30%, and studies have suggested an association between mixed viral infections could increase the disease progression or clinical severity [3].

The most common etiology of ARI worldwide includes influenza virus (InfV), respiratory syncytial virus (RSV), rhinovirus (RV), metapneumovirus (MPV), bocavirus (BV), adenovirus (AV), enterovirus (EV), *Mycoplasma pneumonia*e, parainfluenza virus (PIV), coronavirus (CoV) OC43, NL63, 229E, and HKU1 [4]. Influenza virus types A and B are the leading cause of ARI and serious outbreaks worldwide during the winter season [5]. Annual epidemics of influenza virus alone can cause 5–15% ARI globally. According to WHO, 3–5 million severe cases and 250,000–500,000 deaths occur globally due to annual influenza [6].

In tropical and subtropical countries in the South East Asia such as Thailand, Singapore, Malaysia, and China, the etiologic agents associated with ILI have been well characterized. However, epidemiology and etiology for ILI is poorly understood in Nepal. A laboratory-based ILI surveillance system is well established in Nepal and has greatly contributed to outbreak investigation, surveillance, and timely response since the emergence of pandemic influenza in 2009. The influenza viruses circulating in Nepal have many similarities with our neighboring countries and the regions. We reported year-round transmission of influenza with a peak activity during the rainy and winter seasons is similar to Thailand, Northern Vietnam, and Lao PDR [7]. An efficient detection method is of great importance for laboratory diagnosis. Rapid antigen detection, viral cultures, and molecular methods, such as PCR assays, are currently used as the viral detection method [8]. In clinical settings, rapid and reliable PCR assays are particularly helpful in making early decisions regarding management and treatment of the patients. In this study, we attempt to characterize the influenza and other ARI pathogens by the multiplex PCR assay in children with influenza-like illness cases.

## 2. Materials and Methods

### 2.1. Specimen Collection and Processing

This was a laboratory-based descriptive cross-sectional study conducted from October 2015 to February 2016. A total of 394 throat swab specimens were collected from children aged two months to 12 years with symptoms of influenza-like illness (ILI) during the winter season visiting at National Influenza Center (NIC), National Public Health Laboratory, Kathmandu, Nepal. Of the total, 175 throat swab specimens positive for influenza virus were further tested for detection of other respiratory copathogens. Influenza-like illness was defined as an individual with an acute respiratory infection with history of fever (≥38°C), cough, and/or sore throat with onset within the last 10 days [9]. Throat swab specimens were collected in viral transport medium (Copan, Italy) and transported in triple-package containers at a proper temperature (2–6°C). Specimens were processed in biosafety cabinet class II type A2 (Esco, Singapore) for nucleic acid extraction of influenza and other respiratory pathogens at NIC. Real-time PCR assays were performed for detection of respiratory viruses and other pathogens.

The collected data included patient's demographic characteristics (age and sex); geographic location, type, and subtypes of influenza virus confirmed by real-time polymerase chain reaction (rRT-PCR) assay.

### 2.2. Nucleic Acid Extraction and PCR Amplification

Total nucleic acid (RNA/DNA) was extracted from the throat swab using the PureLink™ Viral RNA/DNA mini kit (Invitrogen, Thermo Fisher Scientific, USA) in accordance with the manufacturer's instructions. An internal control (IC) was added to each extraction tube in order to access the quality of extraction at the end of amplification. Finally, nucleic acid was eluted in 50 *µ*l of the elution buffer and stored at −80°C until use. The real-time rRT-PCR assay for respiratory pathogens was performed on 7500 Fast Real-Time PCR System (Thermo Fisher scientific, USA). Extracted nucleic acid was screened by rRT-PCR with Fast Track Diagnostic (FTD) Respiratory pathogens 21 kit (Biomerieux, Luxemburg) following the manufacturer's protocol using five multiplex PCR for viruses: influenza (InfV) type A-B, influenza A/H1N1, rhinovirus (RV), coronavirus (CoV) OC43, NL63, 229E, and HKU1, parainfluenza virus (PIV) type 1–4, metapneumovirus (MPV) A-B, bocavirus (BV), respiratory syncytial virus (RSV) A-B, adenovirus (AV), enterovirus (EV), parechovirus (PeV), and one bacterial pathogen: *Mycoplasma pneumoniae*. Two positive controls for viral and bacterial multiplex PCR assay, internal control (IC), and a negative control (NC) tubes were provided in the kit. Briefly, 10 *µ*l of the extracted nucleic acid was used as a template in each reaction for the FTD Respiratory pathogens 21 multiplex PCR following the manufacturer's instructions. The thermal cycle amplification condition includes reverse transcription for 15 minutes at 42°C, denaturation for 3 minutes at 94°C followed by 40 cycles for 8 seconds at 94°C, and 34 seconds at 60°C. Specimens were determined to be pathogen positive or negative based on the manufacturer's interpretation criteria, and PCR runs were repeated if a positive and negative control result does not meet the interpretation criteria.

### 2.3. Statistical Analysis

Statistical analysis was performed using SPSS version 11.5. Descriptive statistics, frequency, and percent were generated. Age-wise distribution of coinfection cases, influenza single-infection, and coinfection with other pathogens were described.

### 2.4. Ethical Approval

This study was approved by Nepal Health Research Council (reg. no. 180/2015).

## 3. Results

One hundred seventy-five throat swab specimens positive for any influenza virus were tested to identify coinfection with other possible etiology of ARI. Influenza A virus was detected in 120 (68.6%) specimen, of which 25 (20.8%) were influenza A/H1N1 pdm09 and 95 (79.2%) were influenza A/H3 subtype. Similarly, influenza B virus was identified in 55 (31.4%) specimens. Among those, 175 children positive for any influenza A or B virus; RSV A-B, rhinovirus, adenovirus, metapneumovirus A-B, parainfluenza virus 1 and 3, bocavirus, *Mycoplasma pneumoniae*, enterovirus, coronavirus OC43, 229E, and HKU1 were shown to be infected dually with influenza A and B viruses. Likewise, children were coinfected with *Mycoplasma pneumoniae* (Table 1).

In this study, influenza-positive children cases (*n* = 175) were divided into three subgroups of age and the positive detection rate of respiratory pathogens, and results corresponding to less than 5 years, 6 to 10 years, and 10 to 12 years age groups are shown in Tables 2 and 3. Coinfection with multiple respiratory pathogens in influenza-positive cases was predominant in those younger than five years of age followed by 6–10 and 10–12 years, respectively (Tables 2 and 3).

The proportion of coinfections with rhinovirus 10 (5.7%), respiratory syncytial virus A/B 8 (4.6%), adenovirus 9 (5.1%), and CoV-HKU1 7 (4.0%) viruses was more common in less than five-year-old children with influenza A compared to influenza B-positive cases. Similarly, the rate of coinfections with rhinovirus 6 (3.4%), respiratory syncytial virus A/B 4 (2.3%), adenovirus 3 (1.7%), and CoV-HKU1 2 virus (1.1%) was comparatively found lower among 6–10 year-old children with influenza A and influenza B, respectively (Tables 2 and 3).

Similarly, 38 (21.7%) influenza-positive specimens had been coinfected with two respiratory pathogens; 21 (12.0%) specimens contained three respiratory viruses and 9 (5.2%) specimens contained coinfections of four respiratory pathogens (Table 4). The positive detection rate of influenza A was predominantly found in the month of October 2015 to January 2016. Similarly, influenza B infectivity was found peak in the month of February 2016 followed by October 2015, respectively. Besides influenza virus, rhinovirus, adenovirus, and coronavirus (HKU1) were detected throughout the month of October 2015 to February 2016. Respiratory syncytial virus A-B was detected from the month of November 2015 to February 2016, whereas MPV A-B infection was found during the month of November 2015 to January 2016.

Monoinfection of influenza A/H1N1 pdm09, influenza A/H3, and influenza B was 10.3, 29.1 and 21.7%, respectively. Coinfection of influenza A and B with other single pathogen was found in 21.7% cases, double pathogen was found in 12% cases, and three or more pathogens were found in 17.2% cases (Table 4).

## 4. Discussion

Respiratory virus is a major cause of acute respiratory infection, of which influenza is one of the major public health burdens in developed and developing countries like Nepal. In this study, a total of 175 influenza-positive specimens were investigated for detection of potential respiratory pathogens from October 2015 to February 2016. We identified the coinfection of 9 noninfluenza respiratory pathogens with influenza virus. Of them, the rate of coinfection with rhinovirus, RSV A/B, adenovirus, and CoV-HKU1 viruses was higher. A little information is available concerning the prevalence and seasonality of these viruses, mainly in developing countries like Nepal, where the possibilities of carrying out this type of study on a regular basis are unusual.

During the winter season of 2015/16, an increased number of influenza virus cases (44.4%) were detected and all influenza-positive cases were further screened for other respiratory pathogens by rRT-PCR using FTD respiratory pathogens 21 kit. The spectrum of the pathogens and their positivity rate could vary between country to country and over the time [8]. Influenza virus is one of the major causes of respiratory infection in humans and consequences more severe form than the common cold caused by various types of virus in winter [10]. In our previous study, increased numbers of influenza cases were reported during the rainy and winter seasons of Nepal [7]. Similar findings were reported in Shandong Province, China [11], Bhutan [12], Indonesia [13], and Thailand [14].

Rhinovirus, RSV A-B, adenovirus, and CoV-HKU1 viruses were most frequently detected as coinfecting pathogens in influenza-positive specimens. Similar to our findings, a study conducted by Koul et al. has shown that influenza and rhinovirus were most commonly detected in respiratory samples [15]. However, RSV has been considered as a main etiologic agent of upper respiratory tract infection and pneumonia in children [16] even though rhinoviruses were most frequently detected. In our study, CoV-OC43, CoV-229E, MPV A-B, bocavirus, enterovirus, and parainfluenza virus 1 and 3 coinfections were found in children infected with influenza, which is underreported in Nepal.

Our study detected bocavirus, coronavirus, and enterovirus coinfections in influenza-positive cases. Most deaths from pneumonia in children less than 5 years of age occur in developing countries, where information about the clinical impact and severity of viral causes of respiratory infections is limited [17]. There is a little information on the viral etiology of severe pneumonia in low-income countries, where the disease burden is particularly high [18]. With few exceptions, there is a limited knowledge of bocavirus, coronavirus, and enterovirus and also a lack of data on infection risk factors [19] in Nepal.

Influenza coinfected with *Mycoplasma pneumoniae* (2.9%) was found in influenza-positive children, which is an important pathogen of ARI and community-acquired pneumonia in children [20]. In our study, the majority of the viral infections were found in younger children less than 5 years of age similar to the previous report [21]. Further, incidence of ARIs is especially high among infants, children, and elderly and is more pronounced in low- and middle-income countries [22]. The prevalence of respiratory pathogens in this study is lower than that reported by Islam and his colleagues in India where the prevalence of ARI was found to be 26.2% [23].

In this study, respiratory pathogens could be categorized into four groups: single-infection of influenza A (39.4%), influenza B (21.7%), influenza, and multiple coinfections with two pathogens (21.7), three pathogens (12.0%), and four pathogens (5.2%). Studies conducted in Vietnam [24], Lao PDR [25], Japan [26], the Netherlands [27], and India [28] has reported both single and multiple respiratory infections were found more frequently in young children (<5 year). Similar findings of single and multiple pathogen coinfections with influenza have been reported from Brazil, Turkey, Japan, and China [29–34].

To the best of our knowledge, this could be the first study undertaken with multiplex RT-PCR kit on 21 respiratory pathogen for detection of influenza A, A/H1N1, influenza B, RV, RSV A-B, PIV 1–4, coronavirus OC43, NL63, 229E, and HKU1, MPV A-B, BV, *Mycoplasma pneumoniae*, AV, EV, and PeV. Recently, Rutvisuttinunt et al. have reported the detection rate of viral pathogens (71.3%) in specimens of ARI cases collected from South East Asian countries including Nepal. However, the study had covered nine viral pathogens in specimens contributed by Nepal [35]. Another study conducted in Western Region of Nepal had reported RSV, influenza A-B, PIV-3, *S. pneumoniae,* and *H. influenzae* by assessing the hospital database system; however, the author did not mention the confirmatory method of detection [36].

An increased level of multiple pathogens coinfection with a wide range of respiratory viruses was detected in influenza-positive cases. To the best of our knowledge, findings are new and it was not expected in advance. This study has demonstrated that the wide range of respiratory pathogens is responsible for coinfection in influenza-positive cases in Nepal. However, nothing can be said about the proportion or incidence of other viral infections than influenza as this study was done in a selected group of influenza-positive children. The implication of these findings should be carefully considered in clinical diagnosis and management. In addition, the impact of mono versus multiple coinfections of respiratory pathogens in relation to the severity of disease urges for a complete study in future.

There were several limitations of this study such as very short study period, limited number of samples, and diagnostic reagents. Because of financial constraints, we could not continue our study throughout the year. Hence, the finding of this study does not reflect the whole year scenario. Also, our study could not explore clinical pictures in detail, for example, the severity of illness, period of hospital stay, and its outcome among the single and multiple coinfections, which demands a comprehensive study in future.

Our findings gave baseline information of respiratory viruses and the distribution pattern within the different age groups of children which would help better therapeutic approaches and effective prevention strategies. Furthermore, meticulous attention should be paid to viral infections in younger children.

## 5. Conclusions

Influenza is one of the leading causes of ARIs in children during the winter season in Nepal. In addition to influenza, nine different respiratory viruses were identified. These findings are expected to give better understanding of respiratory viruses, as well as strategies of appropriate case management and minimizing the use of antimicrobial agents in Nepal.

## Figures and Tables

**Table 1:** Details of respiratory pathogen coinfection in influenza-positive cases.

Pathogen	Influenza A (%) *n* = 83	Influenza B (%) *n* = 23	Total positive (%)
Rhinovirus	22 (12.6)	4 (2.3)	26 (14.8)
RSV A/B	15 (8.6)	4 (2.3)	19 (10.8)
Adenovirus	13 (7.4)	1 (0.6)	14 (8.0)
Metapneumovirus A/B	4 (2.3)	1 (0.6)	5 (2.9)
Bocavirus	2 (1.1)	4 (2.3)	6 (3.4)
*M. pneumoniae*	4 (2.3)	1 (0.6)	5 (2.9)
Enterovirus	5 (2.8)	0 (0.0)	5 (2.9)
Parechovirus	0 (0.0)	0 (0.0)	0 (0)
Parainfluenza virus-1	2 (1.1)	1 (0.6)	3 (1.7)
Parainfluenza virus-2	0 (0.0)	0 (0.0)	0 (0)
Parainfluenza virus-3	1 (0.6)	1 (0.6)	2 (1.1)
Parainfluenza virus-4	0 (0.0)	0 (0.0)	0 (0)
Coronavirus (OC43)	3 (1.7)	2 (1.1)	5 (2.9)
Coronavirus (NL63)	0 (0.0)	0 (0.0)	0 (0)
Coronavirus (229E)	2 (1.1)	0 (0.0)	2 (1.1)
Coronavirus (HKU1)	10 (5.7)	4 (2.3)	14 (8.0)

**Table 2:** Distribution pattern of respiratory pathogen coinfection among children in influenza A-positive cases (*n* = 120).

Pathogens	Age group (year)			Total (%)
	<5 (%)	6–10 (%)	10–12 (%)	
RV	10 (5.7)	6 (3.4)	6 (3.4)	22 (12.6)
RSV A/B	8 (4.6)	4 (2.3)	3 (1.7)	15 (8.6)
AV	9 (5.1)	3 (1.7)	1 (0.6)	13 (7.4)
EV	3 (1.7)	1 (0.6)	1 (0.6)	5 (2.8)
MPV A/B	3 (1.7)	1 (0.6)	0 (0.0)	4 (2.3)
*M. pneumoniae*	2 (1.1)	1 (0.6)	1 (0.6)	4 (2.3)
BV	1 (0.6)	1 (0.6)	0 (0.0)	2 (1.1)
PIV-1	1 (0.6)	1 (0.6)	0 (0.0)	2 (1.1)
PIV-3	1 (0.6)	0 (0.0)	0 (0.0)	1 (0.6)
CoV-OC43	2 (1.1)	1 (0.6)	0 (0.0)	3 (1.7)
CoV-229E	1 (0.6)	1 (0.6)	0 (0.0)	2 (1.1)
CoV-HKU1	7 (4.0)	2 (1.1)	1 (0.6)	10 (5.7)

RV, rhinovirus; RSV A/B, respiratory syncytial virus A-B; AV, adenovirus; EV, enterovirus; MPV A/B, metapneumovirus A-B; *M. pneumoniae*, *Mycoplasma pneumonia*; BV, bocavirus; PIV-1, parainfluenza virus-1; PIV-3, parainfluenza virus-3; CoV-OC43, coronovirus-OC43; CoV-229E, coronavirus-229E; CoV-HKU1, coronavirus-HKU1.

**Table 3:** Distribution pattern of respiratory pathogen coinfection among children in influenza B-positive cases (*n* = 55).

Pathogen	Age group (year)			Total (%)
	<5 (%)	6–10 (%)	10–12 (%)	
RV	3 (1.7)	1 (0.6)	0 (0.0)	4 (2.3)
BV	2 (1.1)	1 (0.6)	1 (0.6)	4 (2.3)
RSV A/B	2 (1.1)	1 (0.6)	1 (0.6)	4 (2.3)
AV	1 (0.6)	0 (0.0)	0 (0.0)	1 (0.6)
MPV A/B	1 (0.6)	0 (0.0)	0 (0.0)	1 (0.6)
*M. pneumoniae*	1 (0.6)	0 (0.0)	0 (0.0)	1 (0.6)
PIV-1	1 (0.6)	0 (0.0)	0 (0.0)	1 (0.6)
PIV-3	1 (0.6)	0 (0.0)	0 (0.0)	1 (0.6)
CoV-OC43	2 (1.1)	0 (0.0)	0 (0.0)	2 (1.1)
CoV-HKU1	2 (1.1)	1 (0.6)	1 (0.6)	4 (2.3)

RV, rhinovirus; BV, bocavirus; RSV A/B, respiratory syncytial virus A-B; AV, adenovirus; MPV A/B, metapneumovirus A-B; *M. pneumoniae*, *Mycoplasma pneumoniae*; PIV-1, parainfluenza virus-1; PIV-3, parainfluenza virus-3; CoV-OC43, coronovirus-OC43; CoV-HKU1, coronavirus-HKU.

**Table 4:** Details of pathogens in single and multiple respiratory infections with influenza-positive cases.

Viruses	Positive number (%)
Influenza A/H1N1 pdm09	18 (10.3)
Influenza A/H3	51 (29.1)
Influenza B	38 (21.7)
*Total monoinfection*	**107 (61.1%)**
pdm09 + HPIV1	2 (1.1)
pdm09 + RSV A/B	3 (1.7)
pdm09 + MPV A/B	1 (0.6)
A/H3 + MPV A/B	1 (0.6)
A/H3 + *M. pneumoniae*	1 (0.6)
A/H3 + RSV A/B	7 (4)
A/H3 + AV	4 (2.3)
A/H3 + EV	1 (0.6)
A/H3 + RV	3 (1.7)
A/H3 + HKU1	4 (2.3)
B + BoV	3 (1.7)
B + HKU1	1 (0.6)
B + RV	1 (0.6)
B + *M. pneumoniae*	1 (0.6)
B + MPV A/B	2 (1.1)
B + RSV A/B	3 (1.7)
*Total coinfection with 2 pathogens*	**38 (21.7%)**
pdm09 + RV + EV	1 (0.6)
A/H3 + RV + AV	6 (3.4)
A/H3 + RV + COR43	3 (1.7)
A/H3 + RV+ BV	1 (0.6)
A/H3 + RV + RSV A/B	2 (1.1)
A/H3 + HKU1 + PIV-3	1 (0.6)
A/H3 + RV + EV	1 (0.6)
B + BoV + AV	1 (0.6)
B + HKU1 + PIV1	1 (0.6)
B + HKU1 + RSV A/B	1 (0.6)
B + RV + COR43	2 (1.1)
B + HKU1 + PIV-3	1 (0.6)
*Total coinfection with 3 pathogens*	**21 (12.0%)**
pdm09 + HKU1 + *M. pneumoniae* + RSV A/B	1 (0.6)
A/H3 + HKU1 + *M. pneumoniae* +AV	1 (0.6)
A/H3 + HKU1 + COR229 + PIV1	1 (0.6)
A/H3 + HKU1 + *M. pneumoniae* + RSV A/B	2 (1.1)
A/H3 + RV+ MPV A/B + EV	1 (0.6)
A/H3 + RV + COR229E + RSV	1 (0.6)
H3 + RV + BV + AV	1 (0.6)
H3 + RV + MPV + EV	1 (0.6)
*Total coinfection with 4 pathogens*	**9 (5.2%)**

## Data Availability

The data used to support the findings of this study are included within the article.
